# Flood-conditioned place aversion as a novel non-pharmacological aversive learning procedure in mice

**DOI:** 10.1038/s41598-018-25568-5

**Published:** 2018-05-08

**Authors:** Koral Goltseker, Segev Barak

**Affiliations:** 1 School of Psychological Sciences, Tel Aviv University, Tel Aviv, Israel; 2 Sagol School of Neuroscience, Tel Aviv University, Tel Aviv, Israel

## Abstract

The place conditioning paradigm is an efficient, widely-used method to study mechanisms that underlie appetitive or aversive learning and memory processes. However, pharmacological agents used to induce conditioned place preference (CPP) or aversion (CPA) can *per se* interfere with learning and memory processing, hence confounding the results. Therefore, non-pharmacological place conditioning procedures are of high importance. Here, we introduce a novel procedure for induction of CPA in mice, by water flooding. We found that pairing a context with immersion in moderately cold shallow water resulted in aversion and avoidance of that context during a place preference test. Importantly, place aversion emerged only when mice experienced the onset of flood during conditioning training, but not when mice were placed in a compartment pre-filled with water. We also found that warm water was not sufficiently aversive to induce CPA. Moreover, CPA was observed after two or three context-flood pairings but not after one or four pairings, suggesting that moderate conditioning intensity produces optimal CPA expression. Thus, flood-induced CPA is a simple, cheap, and efficient procedure to form and measure place aversion memories in mice, using an ethologically-relevant threat.

## Introduction

Place conditioning procedures in rodents were initially developed to establish spatial avoidance behavior in rodents using γ- and X-rays^1^. Since then, place conditioning has been widely adopted as a method to measure the rewarding or aversive properties of different treatments^2,3^. In the typical place conditioning procedure, animals are exposed to a set of adjacent, distinctive compartments. One compartment is repeatedly paired with a motivationally significant event, produced by a drug or another reinforcer. The other, unpaired, compartment is paired with the absence of the reinforcer. Learning is measured in a free-choice test: if the reinforcer is rewarding, animals will show preference to the paired compartment (conditioned place preference, CPP); whereas if the reinforcer is aversive, animals will tend to avoid the paired compartment (conditioned place aversion, CPA).

Besides being a relatively simple screening tool for the reinforcing properties of drugs of abuse^2–4^, place conditioning has also been used to study appetitive and aversive learning and memory processes^5–7^, and motivation^8^. To this end, conditioning is carried out with drugs that elicit well-established rewarding (e.g., cocaine, amphetamine) or aversive (e.g., lithium chloride, LiCl) effects^3,5,8,9^. However, besides their motivational effects, pharmacological agents can interfere with neurobiological processes involved in learning and memory^10–12^, raising a confounding issue that complicates interpretation of data acquired in this paradigm. Therefore, establishment of non-pharmacological place conditioning procedures is of high importance. However, while CPP can be induced by a wide range of natural rewards, such as food^8^, sexual and social interaction^13,14^, aggression^15^, and others, there are only a few non-pharmacological approaches to induce CPA.

For example, CPA can be induced by pairing a context with electric foot-shocks^16–18^. However, foot-shocks are non-ethological, noxious stimuli^19,20^. Hence, when a foot-shock is paired with a neutral stimulus, such as an environmental context, the conditioning process fails to predict neural systems that underlie responses to natural threats^21^. In addition, this method requires a customized apparatus with electrified grid-floor and shockers, leading to high costs. Notably, an attempt to establish CPA using a natural threat source, predator odor, yielded only a moderate conditioned response^22^.

Here, we took advantage of the mouse natural reluctance to stay in water, to establish a novel non-pharmacological CPA procedure, by pairing a context with the aversive experience of flooding the compartment with shallow water.

## Results

### Place conditioning in a context flooded with water, but not in a context pre-filled with water, results in CPA

#### Experiment 1

**Figure 1:**
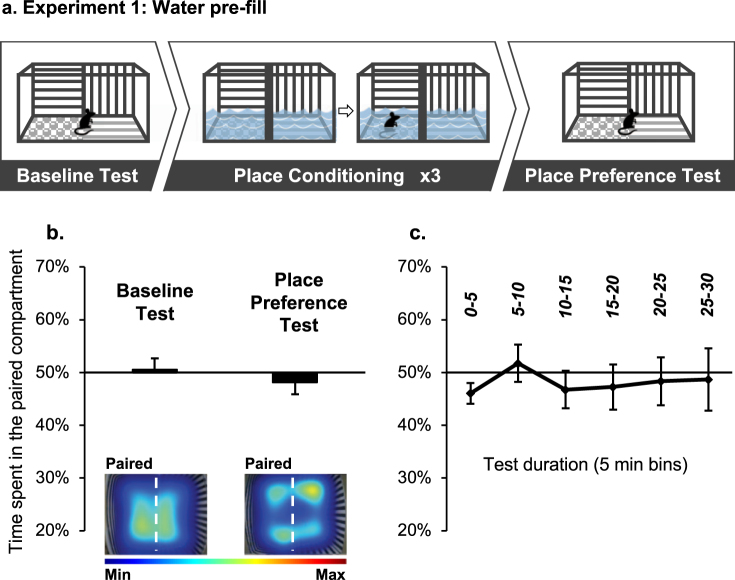
Place conditioning in a context pre-filled with water fails to induce conditioned place aversion (CPA). (**a**) Schematic representation of the experimental procedure. During a Baseline Test mice freely explored the 2-compartment apparatus. Next, during Place conditioning, mice were submitted to a compartment pre-filled with water. On intermittent days, mice were placed in the opposite compartment, which was kept dry during the trial. After 3 pairings with water, mice were given a Place Preference Test. (**b,c**) Place preference/aversion scores, expressed as mean +/− SEM of the percent of time spent in the water-paired compartment, pre-filled with water. (**b**) Preference during the entire baseline and test sessions (30 min); Representative heat maps depict mice location during the Baseline and Place Preference tests. Heat map scale bar represents the normalized time spent at each XY coordinate during the tests. (**c**) Mice performance during the Place Preference test at 5-min temporal resolution. n = 8 per group.

First, we determined whether confining mice to shallow moderately cold water would induce avoidance of the water-paired compartment (Fig. 1; top panel). On the first training day, the sliding door was retracted, and mice could explore the entire dry apparatus freely (Baseline Test). Over the subsequent 6 days, place conditioning was conducted with the sliding door closed. On the 3 *Paired* days, mice were placed in the paired compartment pre-filled with water (18–20 °C; 2 L; ~2 cm depth, reaching the mouse lower body) for 15 min. On the 3 alternate *Unpaired* days, mice were submitted to the unpaired context that remained dry throughout the session. Place Preference Test was performed 24 h after the last conditioning session, and it was identical to the Baseline Test. Place aversion was defined as a decrease in the percent of time spent in the flood-paired compartment during the Place Preference Test, compared to the Baseline Test.

We found that mice spent an equal amount of time in the water-paired compartment during the Baseline and the Place Preference tests, indicating that mice did not express CPA (Fig. 1; t(7) = 0.98, p > 0.05). This finding may suggest that mice did not associate the paired context with the aversive experience of staying in shallow water. Alternatively, it is possible that the shallow water experience was not aversive enough.

#### Experiment 2

**Figure 2:**
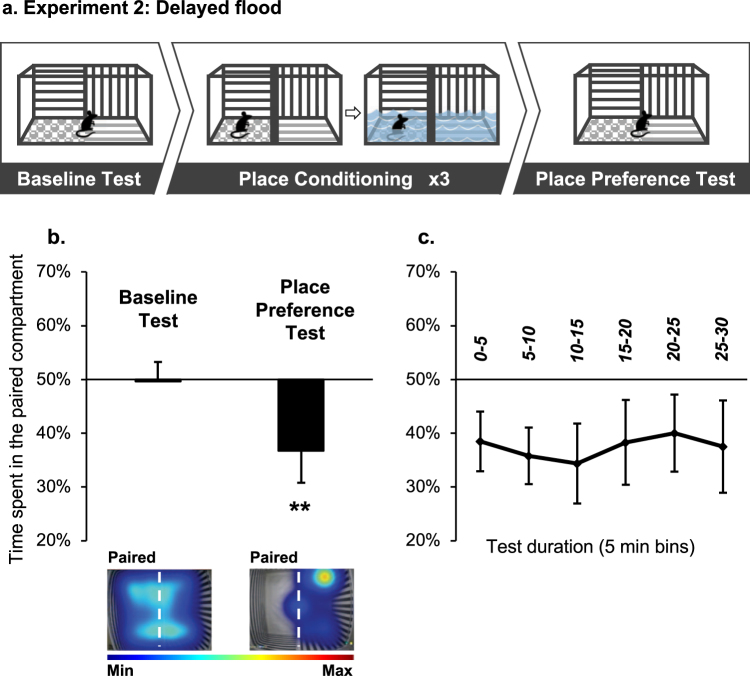
Place conditioning in a context flooded with water results in conditioned place aversion (CPA). (**a**) Schematic representation of the experimental procedure. During a Baseline Test mice freely explored the 2-compartment apparatus. Next, during Conditioning mice were submitted to a dry compartment, gradually flooded with water (Experiment 2). On intermittent days, mice were placed in the opposite compartment, which was kept dry during the trial. After 3 pairings with water, mice were given a Place Preference Test. (**b,c**) Place preference/aversion scores, expressed as mean +/− SEM of the percent of time spent in the water-paired compartment, pre-filled with water. (**b**) Preference during the entire baseline and test sessions (30 min); Representative heat maps depict mice location during the Baseline and Place Preference tests. Heat map scale bar represents the normalized time spent at each XY coordinate during the tests. (**c**) Mice performance during the Place Preference test at 5-min temporal resolution. n = 15 per group; **p < 0.01.

To test a different conditioning strategy, mice were trained in the procedure as described in Experiment 1, except that on the *Paired* days, mice were initially placed in a dry compartment during the 3 water-conditioning sessions (Fig. 2, top panel). Two liters of water (18–20 °C) were poured into the opposite (unpaired) compartment 1–4 min after the session started. As a result, water flowed under the sliding door and gradually flooded the paired compartment (where the mouse was located) to ~2 cm height. Mice remained in the compartment for a total duration of 15 min. Conditioning in the unpaired compartment was as described in Experiment 1.

We found that during the Place Preference Test mice spent less time in the water-paired compartment compared to the Baseline Test, indicating the expression of CPA (Fig. 2; t(14) = 3.36, p < 0.01). Our findings suggest that in this procedure, mice express CPA only if experiencing the onset of the flood, but not when they are placed in a compartment already filled with water.

### Water temperature and conditioning strength modulate the induction of flood-CPA

#### Experiment 3

**Figure 3:**
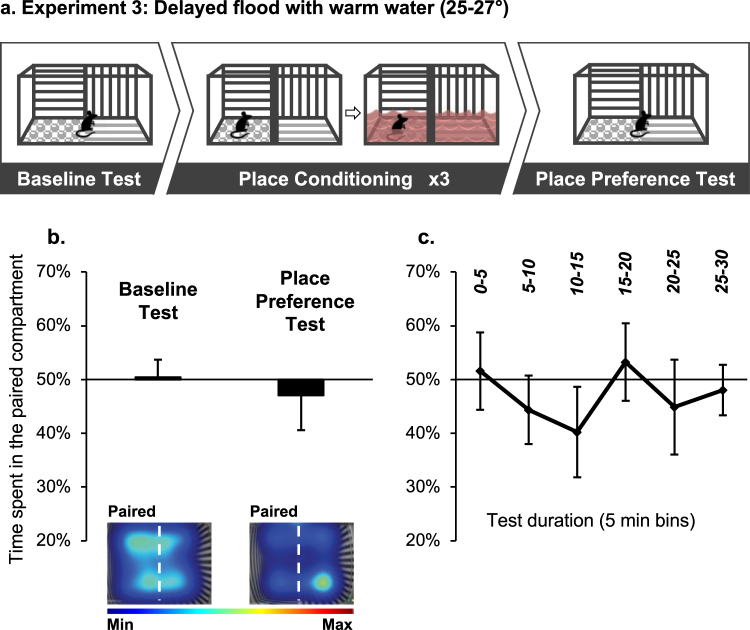
Place conditioning in a context flooded with warm water does not induce conditioned place aversion (CPA). (**a**) Schematic representation of the experimental procedure. During a Baseline Test mice freely explored the 2-compartment apparatus. Next, during Conditioning mice were submitted to a dry compartment, gradually flooded with warm water (25–27 °C; Experiment 3). On intermittent days, mice were placed in the opposite compartment, which was kept dry during the trial. After 3 pairings with water, mice were given a Place Preference Test. (**b,c**) Place preference/aversion scores, expressed as mean +/− SEM of the percent of time spent in the water-paired compartment, pre-filled with water. (**b**) Preference during the entire baseline and test sessions (30 min); Representative heat maps depict mice location during the Baseline and Place Preference tests. Heat map scale bar represents the normalized time spent at each XY coordinate during the tests. (**c**) Mice performance during the Place Preference test at 5-min temporal resolution. n = 8 per group.

To further characterize the parameters critical for this new CPA procedure, we next tested whether CPA could be induced by pairing the compartment with warm water (25–27 °C). Mice were trained as described in Experiment 2 (Fig. 3, top panel). We found that mice spent an equal amount of time in the water-paired compartment during the Baseline and the Place Preference tests (Fig. 3; t(6) = 0.49, p > 0.05). Thus, it is likely that flooding with warm water was not aversive enough to induce avoidance of the water-paired compartment.

#### Experiment 4

**Figure 4:**
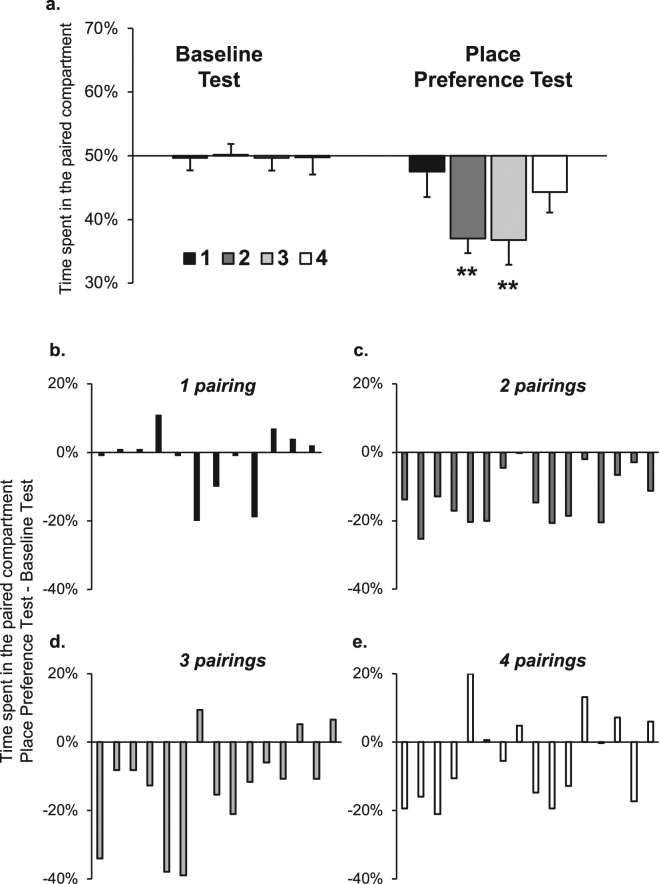
Moderate strength of conditioning is required to establish flood-CPA. (**a**–**e**) Place preference/aversion scores, expressed as mean +/− SEM of the percent of time spent in the water-paired compartment following 1, 2, 3 or 4 conditioning sessions. (**b**–**e**) Change in the place preference scores, expressed as the percent of time spent in the water-paired compartment during the Place Preference Test minus the Baseline Test. Bars represent individual data from mice that underwent 1 (**b**), 2 (**c**), 3 (**d**), or 4 (**e**) context-flood pairings. n = 12–16 per group, **p < 0.01.

Next, we determined the optimal number of conditioning sessions to establish water-CPA. The procedure was as described in Experiment 2, except that groups differed in the number of place conditioning sessions (Fig. 4a). Overall, we found that water-flood place conditioning led to strong CPA (main effect of Test: F(3,55) = 29.68, p < 0.0001; Test X Pairings interaction: F(3,55) = 3.12, p < 0.05). Further analysis revealed that while 1 and 4 pairings with water did not produce a significant CPA effect (p’s > 0.1), 2 and 3 pairings resulted in the expression of CPA (p’s < 0.01). These findings form an (inverted) U-shape curve of place aversion as a function of conditioning strength, with weak and strong conditioning not producing effective CPA.

## Discussion

We introduce here a novel behavioral (non-pharmacological) method to induce CPA in mice. We show that pairing a context with a flood of moderately cold shallow water results in avoidance of that context during a place preference test. Importantly, we show that place aversion emerges only when mice experience the flood of water in the compartment during a conditioning session, but not when placed in the compartment pre-filled with water. Moreover, we show that warm water is not sufficiently aversive to induce CPA. Finally, we show that CPA is evident after 2 or 3 context-flood pairings, but not after 1 or 4 pairings, suggesting that moderate conditioning intensity produces optimal CPA expression.

Behavioral manipulations involving full or partial immersion of rodents in water are considered highly aversive, and have been used to induce stress^23,24^, to assess depression-like behavior^25^ and to test aversively motivated spatial learning^26,27^. Notably, unlike the procedure described here, in well-established aversive tasks such as the forced swimming test^25^ or the Morris water maze^26^ rodents are confined to deep water and are bound to swim or float during the session. Nevertheless, confinement to shallow water is an established aversive event^23,28,29^, and has been shown to generate escape motivation in a paddling water maze^28^, and to reduce the number of the pool-crossings for food^30^.

Our finding that CPA was exhibited in cool, but not in warm water, supports the notion that low water temperature can motivate animals to learn^27,31,32^. Specifically, training in moderately cold water (18–20 °C), as compared to warm water (25–27 °C), has been found to increase the rate of acquisition of escape behavior in the Morris water maze^32,33^, in a radial-arm water maze^34^, in a water T-maze^27^, and in a paddling maze^31^. The modulatory effect of water temperature in learning procedures has been attributed to higher levels of corticosterone detected in animals trained at 19 °C *versus* 25 °C^32,33^. Indeed, corticosterone has been previously shown to enhance memory formation, if induced during the learning procedure^35,36^. Therefore, our procedure is a potential non-pharmacological tool to study the naturally occurring physiological processes underlying the memories of aversive, threatful events.

We found that CPA was expressed only when mice were allowed to briefly explore the dry surrounding at the beginning of the conditioning sessions, before water flooded the paired compartment. In contrast, mice did not express CPA when placed in the paired compartment pre-filled with water. The experience of water (unconditioned stimulus; US) flooding the dry paired compartment (conditioned stimulus; CS) is a form of forward conditioning, in which the presentation of the CS precedes the onset of the US. In contrast, the pre-filled setting corresponds to simultaneous conditioning, in which the CS and US are presented simultaneously, or possibly to a form of backward conditioning, as the plunge into water might precede exploration and identification of the paired contextual environment. Thus, our results are in concordance with previous findings, suggesting that forward conditioning is significantly more effective in producing a conditioned response (here, CPA) compared to simultaneous or backward conditioning^37–39^.

Alternatively, the lack of CPA expression in the pre-filled setting, may imply that the presence of shallow water during the entire conditioning session became a component of a contextual CS, which signaled an aversive event. Thus, in the absence of water during the Place Preference test, mice failed to express CPA, because the CS presented in the test was different from the CS in the conditioning phase.

Interestingly, we found that only moderate strength of conditioning, namely, 2–3 conditioning sessions, produced CPA, whereas weaker or stronger conditioning (1 or 4 conditioning sessions) failed to produce CPA, forming an (inverted) U-shape conditioning-response curve. Indeed, a single conditioning session has previously been reported insufficient to produce CPP or CPA^6,40^, whereas an increase in the number of pairings (4 and more) usually results in a stronger conditioned response^2,6^. However, there is also evidence that CPA, induced by a few context-drug pairings, can be reduced and even reversed to CPP following repeated/extended conditioning, due to the development of tolerance to the adverse effect of the reinforcer, and to the emergence of its rewarding properties^2,41^. Similarly, the absence of CPA expression after 4 pairings in our study might suggest that mice gradually habituated to the shallow-water manipulation. In line with this assumption, behavioral^42^ and somatosensory^43^ stress responses were reported to decay steadily following repeated exposures to a mild stressor. Hence, it is possible that extended conditioning resulted in perceiving the water flood as less aversive for some mice, thus weakening the overall CPA effect acquired during the first three sessions. Nevertheless, using the water-flood CPA procedure in the future will require careful choice of parameters, with particular emphasis on the number and duration of conditioning sessions.

Overall, flood-CPA is a promising tool to explore conditioned aversive responses and their neural substrates. To date, the CPA phenomenon has been mostly studied by pairing a context with the emetic effects of LiCl^9,17,44–47^. However, the interpretation of LiCl-CPA data can be inconclusive, as LiCl *per se* was shown to interfere with neuronal mechanisms involved in memory processing, such as NMDA receptor^11^ and glycogen synthase kinase 3β (GSK3β)^48^ signaling. As a non-pharmacological alternative, CPA can be induced by pairing one of the spaces of a 2- or 3-compartments chamber with footshocks^16–18^. However, such non-ethological, noxious stimuli^19,20^ might not implicate neural systems that underlie responses to natural threats^21^. Moreover, this method is rarely applied due to the equipment complexity. Interestingly, pairing a context with a predator odor (trimethylthiazoline (TMT), a component of fox feces), produced only weak CPA^22^, in line with previous indications that TMT’s aversive potential is not robust enough to induce extensive conditioned responses^49,50^. Thus, our present findings provide a unique and simple new behavioral method to induce robust CPA without confounding pharmacological effects.

In summary, we introduce here a novel, non-pharmacological CPA procedure, by pairing a neutral context with an ethologically relevant threatful event, flooding by water. Flood-CPA is a simple, non-expensive procedure to form and measure aversive contextual learning and memory in mice. Moreover, it is an efficient alternative to procedures based on pharmacological treatment or electrical foot-shock, to study the neurobiology of aversive learning and memory processes.

## Methods and Materials

### Animals

Male and female C57BL/6 J mice (bred at Tel-Aviv University Animal Facility, Israel; 25–30 g) were housed 3–4/cage and kept under a 12-h light-dark cycle (lights on at 4 a.m.) with food and water available *ad libitum*. All experimental protocols were approved by, and conformed to, the guidelines of the Institutional Animal Care and Use Committee of Tel Aviv University, and to the guidelines of the NIH (animal welfare assurance number A5010-01). All efforts were made to minimize the number of animals used.

### Apparatus

All experiments were performed in Plexiglas boxes (30 × 30 × 20 cm) separated non-hermetically into two equal-sized compartments by a white plastic sliding door. The compartments differed from each other by the wall pattern (horizontal vs. vertical b/w stripes; 1 cm width) and the floor surface (white textured plastic; with bulging circles vs. bulging stripes). The horizontal stripes pattern on the walls was always matched with the bulging circles on the floor, whereas the vertical stripes – with the bulging stripes. The ceiling was covered with a removable transparent Plexiglas sheet to prevent mice from escaping. Each Plexiglas box was placed in a sound-attenuating chamber equipped with a LED light stripe on the walls and a ceiling camera that registered mouse behavior. Data were recorded by Ethovision XT 11 (Noldus, Wageningen, Netherlands).

### Place conditioning procedure

All mice were habituated to handling for 3 days prior to the beginning of the procedure. The procedure consisted of 3 stages: *Baseline Test*. On the first day, the sliding door was retracted, and mice could explore the entire dry apparatus freely for 30 min. Animals that spent >70% of time in either of the compartments were excluded from the study. This allowed the use of an unbiased design, in which the 2 compartments are: a) equally preferred before conditioning, as indicated by the group average (unbiased apparatus); b) pseudo-randomly assigned to the experimental conditions (unbiased assignment procedure)^3^. *Place conditioning*. Conditioning started 24 h after the Baseline Test with one session per day, with the sliding door closed. On *Paired* days, one of the compartments was paired with immersion in shallow water for 15 min. Procedurally, 2 liters of water were poured by the experimenter into the unpaired compartment. As a result, water flowed under the non-hermetic sliding door and gradually flooded the paired compartment to ~2 cm height. Thus, the entire conditioning box remained filled with water throughout the conditioning session; however, the sliding door restricted the passage between compartments.

In Experiment 1, mice were submitted to the paired compartment pre-filled with water, in Experiments 2–4, mice were submitted to a dry paired compartment, flooded with water 1–4 min later, as described above. After water-conditioning trials, mice were placed in a cage with dry bedding (collected from their home cage, to prevent a competing conditioning of a novelty pairing^51,52^), and then returned to the home cage, when dry. Conditioning boxes were emptied and dried between trials. On the alternate, *Unpaired* days, mice were confined to the dry unpaired compartment for 15 min. Paired compartments were counterbalanced. *Place Preference Test*. The test was performed 24 h after the last conditioning session, and was identical to the Baseline Test. Place aversion was defined as a decrease in the percent of time spent in the flood-paired compartment on the Place Preference Test day, compared to the Baseline Test.

### Data analysis

Data were analyzed by a paired t-test, or by a mixed-model two-way ANOVA with a between-subjects factor of Pairings, and a repeated measures factor of Test. ANOVA was followed by a Student–Newman–Keuls post hoc test. Sex distributed approximately equally across experiments, and was initially analyzed as a factor; however, all analyzes did not yield a main effect of sex or any interaction with other factors (p’s > 0.05). Therefore, data were collapsed across this factor. Two mice were excluded from the analysis of Experiment 4 due to their biased preference of one of the compartments prior the conditioning phase (>70% of time in either of the compartments during the Baseline Test).

### Experimental design

#### Experiment 1: Place conditioning with a compartment pre-filled with water

On Day 1, mice underwent a free-choice Baseline Test. Place conditioning was conducted on Days 2–7. On Days 2, 4, and 6, mice were confined to a dry unpaired compartment. On Days 3, 5, and 7, mice were submitted to the paired compartment pre-filled with water (18–20 °C). Place Preference Test was conducted on Day 8.

#### Experiment 2: Place conditioning with a delayed flood

Training schedule and procedure were similar to Experiment 1, with the exception that on Days 3, 5, and 7, mice were submitted to a dry paired compartment, which was filled with water (18–20 °C) 1–4 min later. The effect of the delayed water-flood was evaluated in two replications that produced similar results, therefore data were pooled together.

#### Experiment 3. Place conditioning with warm-water flood

Mice underwent place conditioning as in Experiment 2, except that we used warm water (25–27 °C).

#### Experiment 4. Intensity of flood-place conditioning

Training was similar to Experiment 2, with different number of conditioning sessions. Thus, mice received 1, 2, 3 or 4 compartment-flood pairings, after which they were tested for place preference. The effect of 4 pairings was re-evaluated in a replication that produced similar results, therefore data were pooled together.
